# The PsbS protein and low pH are necessary and sufficient to induce quenching in the light-harvesting complex of plants LHCII

**DOI:** 10.1038/s41598-021-86975-9

**Published:** 2021-04-01

**Authors:** Lauren Nicol, Roberta Croce

**Affiliations:** Biophysics of Photosynthesis, Department of Physics and Astronomy, Faculty of Sciences, Vrije Universiteit Amsterdam, De Boelelaan 1081, 1081 HV Amsterdam, The Netherlands

**Keywords:** Photosynthesis, Biological fluorescence

## Abstract

Photosynthesis is tightly regulated in order to withstand dynamic light environments. Under high light intensities, a mechanism known as non-photochemical quenching (NPQ) dissipates excess excitation energy, protecting the photosynthetic machinery from damage. An obstacle that lies in the way of understanding the molecular mechanism of NPQ is the large gap between in vitro and in vivo studies. On the one hand, the complexity of the photosynthetic membrane makes it challenging to obtain molecular information from in vivo experiments. On the other hand, a suitable in vitro system for the study of quenching is not available. Here we have developed a minimal NPQ system using proteoliposomes. With this, we demonstrate that the combination of low pH and PsbS is both necessary and sufficient to induce quenching in LHCII, the main antenna complex of plants. This proteoliposome system can be further exploited to gain more insight into how PsbS and other factors (e.g. zeaxanthin) influence the quenching mechanism observed in LHCII.

## Introduction

In plants, light absorption is maximized with specialized light-harvesting complexes (LHCs) that bind a high density of chlorophylls (Chls) and carotenoids. The specific organization of pigments and proteins enables efficient transfer of excitation energy to the Photosystem II (PSII) reaction centre where photosynthesis is initiated with photochemical charge separation.^1^ However, the intensity of natural light can fluctuate rapidly, and the amount of light absorbed often exceeds the capacity of the photosynthetic reactions. A feedback mechanism known as non-photochemical quenching (NPQ) acts as a safety valve, dissipating the excess excitation energy as heat, preventing the formation of Chl triplets and the inevitable production of reactive oxygen species^2^.

NPQ is triggered by an increase in lumen proton concentration and the subsequent protonation and activation of the integral membrane protein, PsbS^3–5^. Although PsbS is a member of the LHC superfamily, it has some unusual characteristics. It is not an integral part of the PSII-LHC supercomplex^6–8^, it is substoichiometric with respect to the PSII core^9^, and its compact four-transmembrane helix fold precludes the binding of pigments^10^. The absence of stably bound pigments suggests PsbS is not the site of quenching; therefore, the current view is that PsbS promotes the formation of quenching sites in the LHCs^11^ or at the interface between LHCs and PsbS^12^. This is supported by the substantial reduction of NPQ capacity in the absence of LHCs in vivo^13^.

The first hypothesis proposes that PsbS is responsible for quenching in LHCs by promoting LHC oligomerization and/or increasing the probability of the quenched conformation. It has long been observed that LHC oligomers are highly quenched in vitro^14,15^. The oligomers (sometimes referred to as aggregates) can be formed via detergent removal^16,17^ or spontaneous clustering in the lipid bilayer of proteoliposomes^18^. Oligomerization is not necessary for LHCs to access the quenched state per se. LHCs can access a variety of spectral and kinetic states via conformational changes in the protein scaffold modifying pigment–pigment interactions^19–23^. An oligomer consists of a heterogeneous mixture of these conformational states, and quenching arises from a small number of complexes in a strongly quenched conformation being energetically connected to the bulk of the unquenched complexes^24,25^. Parallels have been drawn between aggregation-related quenching in vitro and what occurs during NPQ in vivo. For example, increased far-red fluorescence emission, a spectroscopic signature of LHC oligomerization, has been observed in leaves following NPQ induction^26,27^. Furthermore, freeze-fracture electron microscopy of intact plant chloroplasts shows that LHCII has a tendency to cluster during NPQ^28^. This clustering is inhibited in the PsbS knockout mutant and enhanced in the PsbS overexpressor^17,29^.

The second hypothesis proposes a more direct role of PsbS in quenching, via the formation of a quenching site at a PsbS–LHC interface^12,30,31^. The idea is that a pH-dependent conformational change of PsbS, such a monomerization, could allow the transient binding of a pigment present in the thylakoid membrane. The PsbS-pigment complex could then strongly interact with an LHC, positioning the carotenoid in close contact with one of the peripheral Chls, thus creating a quenching site. While the pigment in question has been suggested to be zeaxanthin^12^, it could equally be another pigment with the capacity to quench Chl excited singlet states such as lutein^23,32^.

To further investigate the interaction between PsbS and LHCs, several studies have utilized a proteoliposome system containing PsbS and LHCII^33–36^. LHCII is a trimeric complex, and as the main antenna of PSII is responsible for the majority of NPQ^37^. The advantage of a proteoliposome system is that it avoids the complexity of the thylakoid membrane whilst allowing interprotein interactions within the plane of the membrane. Surprisingly, however, the results of these studies are at variance with what is known to occur in vivo. Most observe quenching at neutral pH suggesting the quenching is an artefact^33–35^, or no quenching at all ^36^. In the current study, we improve upon existing proteoliposomes systems by more closely mimicking the native thylakoid environment. We aim to reproduce NPQ as it occurs in vivo i.e. fluorescence quenching that is both pH- and PsbS-dependent. This will allow determination of the minimal components necessary for NPQ and shed light on the action mechanism of PsbS.

## Results and discussion

### Purification of PsbS from the thylakoid membrane

**Figure 1:**
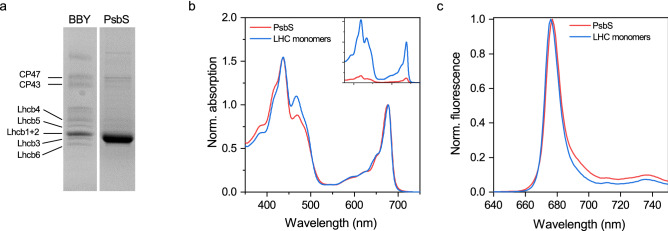
Protein composition and spectral characteristics of the PsbS eluate following strep-tactin affinity chromatography. (**a**) SDS-PAGE of WT BBY membranes and PsbS eluate. Lanes are cropped from different parts of the same gel. The uncropped gel is presented in Supplementary Figure S4. (**b**) absorption spectra of PsbS eluate and LHC monomers normalized to the Q_y_ maximum. The inset shows the absorption spectra normalized to protein concentration. (**c**) 77 k fluorescence emission spectra of PsbS eluate and LHC monomers normalized to the maximum. Results are representative of three independent experiments with similar results.

*Arabidopsis thaliana* PsbS knockout plants were transformed with PsbS modified to contain a strep-tag at the C terminus. These plants have a ~ 50% increase in the amount of qE compared to WT plants and a corresponding ~ 65% increase in the stoichiometry of PsbS to the PSII core, confirming that the NPQ level increases with the increase of the amount of PsbS in the membrane and indicating that the strep-tag has minimal effect on PsbS function (Supplementary Fig. S1). PSII-enriched membranes (BBY) isolated from the mutant plants were solubilized with a mild detergent, and PsbS was isolated in a single step via affinity chromatography. The purity of PsbS in the eluate was determined to be approximately 90% based on SDS-PAGE analysis, and the impurities appear to be a non-specific array of PSII components (Fig. 1a). This is consistent with the highly hydrophobic properties of PsbS, and its tendency to form non-specific interactions with complexes upon membrane solubilisation^38^. Given that PsbS is not a pigment-binding protein, the pigments in the eluate are likely bound to the protein impurities. This is supported by the resemblance of the absorption spectra to LHC monomers (Lhcb1-6; found in band 2 of a sucrose gradient^7^) and the low absorption intensity relative to protein concentration (Fig. 1b). The absence of any strong fluorescence emission in the 720–740 nm range at 77 K, excludes the presence of PSI or LHCI (Fig. 1c).

LHCII was obtained by solubilisation of the thylakoid membrane with α-DM. The preparation has been previously characterized^39^. The complex contains 3.8 carotenoids (0.9 neoxanthin, 0.3 violaxanthin and 2.6 luteins) per 14 Chls (8 Chl a and 6 Chl b). No zeaxanthin was detected in the preparation (Supplementary Fig. S2) in agreement with the fact that the complex was purified from dark-adapted, non-stressed plants.

### LHCII folds correctly in proteoliposomes at neutral and low pH

Liposomes were prepared from native thylakoid lipids, MGDG, DGDG, SQDG and PG in a molar ratio similar to what is found in vivo^40^. The liposomes were destabilized in 0.03% α-DM, and LHCII was added to the liposome preparation in a lipid:protein molar ratio of 133:1. The proteins were incorporated into the lipid bilayer by removing detergent with polystyrene beads. Any unincorporated protein was removed from the final preparation via centrifugation. A decrease in the absorption of the final preparation indicates that LHCII reconstitution efficiency is approximately 70% at pH 7.5 and 40% at pH 5.5 (Supplementary Fig. S2). A trypsin digest revealed that there is no preferential orientation of LHCII in the liposome, so the interaction between complexes with opposite orientation cannot be excluded (Supplementary Fig. S2).

**Figure 2:**
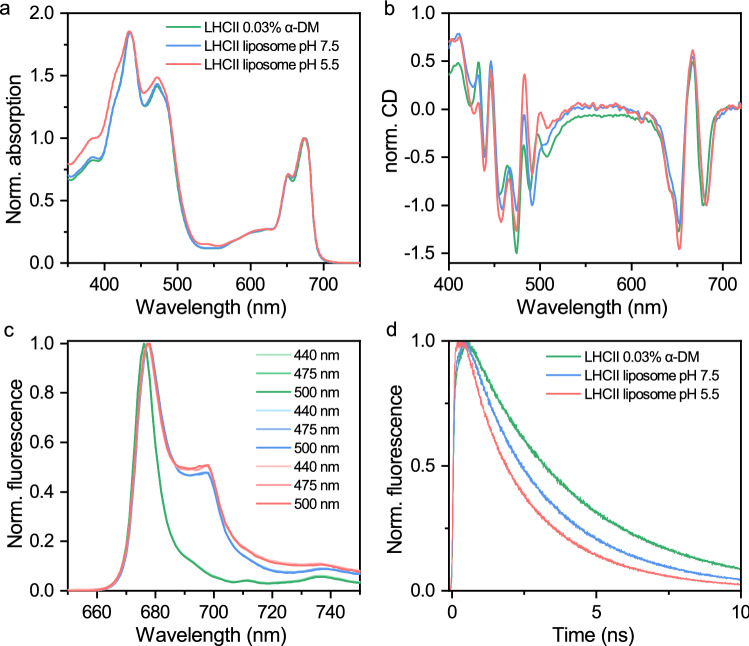
Spectral characteristics of LHCII in detergent (at pH 7.5) compared to LHCII reconstituted into liposomes at pH 7.5 or pH 5.5. (**a**) Absorption spectra normalized to the Q_y_ maximum (**b**) circular dichroism spectra normalized to the negative ~ 680 nm peak (**c**) 77 K emission spectra with excitation wavelengths of 440 nm, 475 nm and 500 nm, normalized to the maximum (**d**) fluorescence decay traces upon excitation at 468 nm and detection at 680 nm. Proteoliposome preparations had lipid:protein molar ratios of 133:1. Results are representative of three independent experiments with similar results.

The resulting proteoliposomes have absorption spectra very similar to LHCII in detergent, demonstrating there is no pigment loss during the procedure (Fig. 2a). The 600–700 nm region of the CD spectra indicate that pigment organization is similar to that of LHCII in detergent (Fig. 2c). Differences in the CD spectra at shorter wavelengths are expected as this region is highly sensitive to protein environment^41^. Overlap of the low-temperature fluorescence emission spectra upon excitation of Chl a (440 nm), Chl b (475 nm) and carotenoids (500 nm) demonstrate efficient excitation energy transfer between pigments and proper folding of complexes (Fig. 2b). Overall, the results confirm correct folding and incorporation of LHCII into the lipid bilayer at both neutral and low pH.

### LHCII is pre-clustered and pre-quenched in the proteoliposome

Bulk LHCII can exist in a continuum between light-harvesting and quenched states. In detergent micelles, LHCII is the fully light-harvesting state, whereas, in the thylakoid membrane, it is poised between light-harvesting and quenched states^42,43^. This partial quenching is a result of protein crowding and LHCII oligomerization, rather than direct lipid-protein interactions^18,44^. In proteoliposomes, the position of LHCII on the continuum can be finely tuned by controlling the lipid:LHCII ratio, where low lipid:LHCII ratios increase the propensity of LHCII to aggregate, resulting in a shift towards the quenched state^18^. The extent of oligomerization can be monitored by the intensity of a low-temperature fluorescence emission peak at ~ 700 nm and the degree of quenching can be monitored directly via the rate of LHCII fluorescence decay^18^.

In the present study, LHCII in the proteoliposome displays a substantial increase in the low-temperature fluorescence emission at ~ 700 nm (Fig. 2c) and a faster rate of fluorescence decay (Fig. 2d) compared to LHCII in detergent. Average fluorescence lifetimes were obtained by fitting fluorescence decay curves to a sum of exponential decay components (Supplementary Table S1). LHCII in detergent has a lifetime of 3.3 ns, whereas LHCII in the proteoliposome prepared at pH 7.5 has an average lifetime of 2.4 ns, similar to the ~ 2 ns lifetime observed in the thylakoid membrane^42^. This is further shortened to 1.6 ns when proteoliposomes were prepared at pH 5.5, supporting the notion that LHCII has some intrinsic pH sensing capacity when in the aggregated state^45^. While this effect is almost negligible in terms of contribution to NPQ in WT plants, it is thought to be the cause of a slowly forming NPQ (on the order of 1 h) in the PsbS knockout mutant^46^. Also note that low pH in the absence of aggregation is insufficient to induce quenching in isolated LHCII^47^.

### PsbS induces quenching in LHCII at low pH

**Figure 3:**
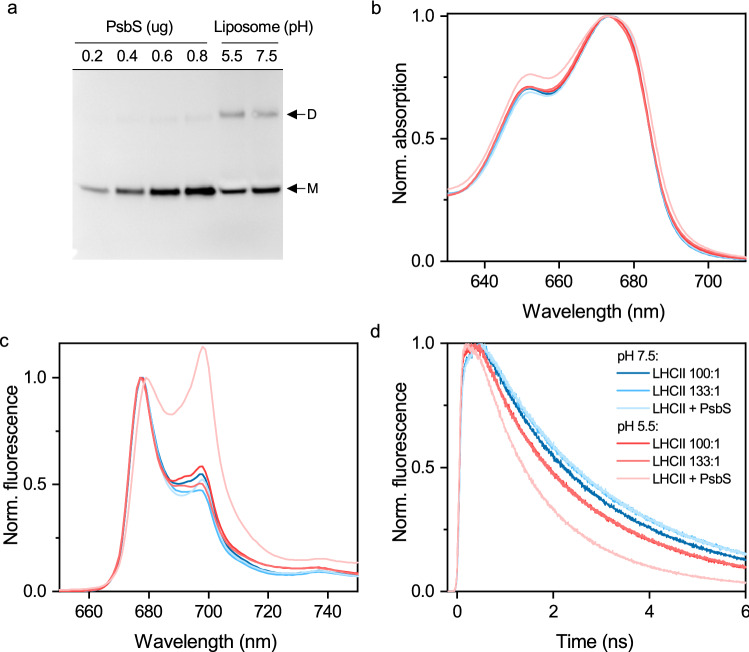
The effect of PsbS on LHCII spectral characteristics in the liposome at pH 7.5 and pH 5.5. (**a**) Immunoblot of PsbS in detergent and in LHCII + PsbS proteoliposomes where M and D denote the monomeric and dimeric forms of PsbS, respectively. The full-length blot is presented in Supplementary Figure S4. (**b**) Absorption spectra normalized to the Q_y_ maximum (**c**) 77 K fluorescence spectra upon excitation at 440 nm, normalized to the maximum. (**d**) Fluorescence decay traces upon excitation at 468 nm and detection at 680 nm. LHCII-only proteoliposome preparations have lipid:protein ratios of 100:1 and 133:1. LHCII + PsbS proteoliposome preparations have total lipid:protein ratios of 100:1, lipid:LHCII ratios of 133:1 and PsbS:LHCII ratios of 1:3. Results are representative of three independent experiments with similar results, which can be viewed in Supplementary Figure S3.

To investigate the PsbS-LHCII interaction, PsbS was added in a 1:1 molar ratio with LHCII trimers. This stoichiometry is higher than what naturally occurs in the thylakoid membrane but is intended to increase the probability of PsbS-LHCII interaction when liposome occupancy is known to be heterogeneous^48^. Immunoblotting confirmed successful reconstitution of PsbS into the liposome, with the lipid bilayer appearing to stabilize the dimeric state (Fig. 3a). The lipid:LHCII ratio of this sample is 133:1, and the total lipid:protein ratio is 100:1. To distinguish the effect of PsbS and increased protein concentration, we have also used an LHCII-only control with a lipid:protein ratio of 100:1. In this instance, the increase in protein concentration did not have a large effect on results (Fig. 3b–d).

The addition of PsbS had no significant impact on LHCII spectral characteristics at pH 7.5; however, at pH 5.5, a number of differences are observed. There is a broadening of the Q_y_ absorption peak and a relative increase in absorption at 650 nm (Fig. 3b), a significant increase in low-temperature fluorescence emission at 700 nm (Fig. 3c) and a shortening of LHCII fluorescence lifetime to 1.1 ns (Fig. 3d). Using proteoliposomes containing PsbS at neutral and low pH as representative of the unquenched (F_m_) and quenched (F_m_') state respectively, we can approximate the level of NPQ obtained in this system is around 1, a value very similar to what is reported for isolated thylakoids and chloroplasts^28,39,49^.

The requirement of both PsbS and low pH to induce quenching shows that our system mimics the properties of NPQ in vivo. This is in contrast to previous PsbS-LHCII proteoliposome systems, where quenching was observed at neutral pH^32–35^. This discrepancy is likely due to the effect of lipid:protein ratio on LHCII lifetime. As mentioned, increasing LHCII concentration in the liposome increases the tendency to cluster and shortens fluorescence lifetime^18^. It is conceivable that increasing the protein concentration via the addition of PsbS could have the same effect, highlighting the importance of adequate protein concentration controls. The lack of additional quenching at low pH in these studies can presumably be attributed to the use of recombinant PsbS, which may have a different tertiary structure to that of PsbS folded in vivo.

The data also demonstrates that a minimal system composed of thylakoid membrane lipids, PsbS, LHCII and low pH is sufficient for non-photochemical quenching. This excludes the possibility that other components of the thylakoid membrane are essential for quenching and discounts the hypotheses that quenching centres are created by the transient association of pigments with PsbS ^12,30,31^. The low pH, PsbS-induced increase in fluorescence emission at 700 nm suggests that PsbS triggers clustering of LHCII. This is fully consistent with the observations of enhanced PsbS-LHCII interactions upon NPQ activation in intact chloroplasts^50,51^ and further supports the role for aggregation-related quenching in vivo^14,17,29^.

It is important to note that red fluorescence emission is not a direct spectroscopic marker of the quencher, but of aggregation. Oligomerized LHCII can exist in a variety of conformational states characterized by different emission spectra^24^. The red-emitting state is relatively long-lived and results from a Chl–Chl charge transfer state that acts as a trap for excitation energy^24,52^. Clustering of LHCII leads to an increase of the population of these forms, an effect that is particularly visible at low temperature. The quenched conformation has been shown to emit at 680 nm^23,24^ where the quencher is a carotenoid dark state, which is populated by excitation energy transfer from neighbouring Chls^23,32^. Whether PsbS increases the probability of this quenched conformation or influences the physical quenching mechanism requires further spectroscopic analysis. This will be aided by the simplicity of the proteoliposome system, which lacks the overlapping spectral and kinetic signals from Photosystems and other LHCs present in the thylakoid membrane.

The flexibility of the system will permit the investigation of mutants of both PsbS and LHCII to identify the pigments/protein domains involved in the quenching process. Furthermore, the addition of zeaxanthin, and/or LHCII binding zeaxanthin in the V1 site will provide valuable insight into zeaxanthin dependent quenching.

## Methods

### Plant growth

*Arabidopsis thaliana* WT (Col-0) and PsbS KO (*npq4*) lines were obtained from the Nottingham Arabidopsis Stock Centre (NASC). All plants were grown under 120 μmol photons m^−2^ s^−1^, 16 h/8 h day/night cycle for 4–5 weeks. The research involving plants complies with relevant institutional and national guidelines and legislation.

### PsbS-strep construction

The PsbS promoter region was amplified by PCR from genomic DNA isolated from *A. thaliana* with the forward primer 5′ TTCAAGCTTAGGGGTTTAATGTATGTACA 3′ and reverse primer 5′ TTCCTCGAGTCTTTCTGAGGATGAGAGAA 3′. The product was cloned into pORE-O3^53^, using HindIII and XhoI restriction sites. The PsbS coding region was amplified by PCR from genomic DNA isolated from A. thaliana with the forward primer 5′ TAGAATTCAAGAATGGCTCAAACCATGCTGCTTACTTCAG 3′ and reverse primer 5′ TAGCGGCCGCTTATTTTTCAAACTGCGGATGGCTCCACGCGCTGCTTTCTTCACCATCATCGG 3′. The PCR product was digested with EcoRI and NotI before re-amplification with the forward primer 5′ AGAAGGCCTTGGATCCAGAATGGCTCAAACCATGCTGCT 3′ and reverse primer 5′ CTAGCGGCCGCTTATTTTTCAAACTGCGGATGGCTC 3′. The reverse primer was designed to add a two amino acid spacer SA and a tail of WSHPQFEK, which constitutes the strep-tag. The product was cloned into pORE-O3 containing the PsbS promoter, using BamHI and NotI restriction sites. *Agrobacterium tumefaciens* strain GV3101 was transformed with sequence confirmed constructs. PsbS KO (*npq4*) plants were transformed by floral dip^54,55^. Transformants were selected with the herbicide glufosinate. Expression of the PsbS transgene was assessed by immunoblotting, and a homozygous line was identified in subsequent generations. Thylakoids were prepared according to^39^. PsbS activity was confirmed by measuring NPQ of leaves in a modular Dual PAM-100 apparatus (Walz) as in^37^. NPQ is calculated as (F_m_ − F_m_) · F_m_^−1^.

### Protein isolation

Grana-enriched membranes (BBY) were prepared from WT and PsbS-strep plants as in^7^. WT BBY were used to prepare LHCII trimers according to ref^7^ and HPLC was performed as in^39^ to ensure the retention of ≥ 3.8 xanthophylls per 14 Chls (Supplementary Figure S2). PsbS-strep BBY were unstacked in 5 mM EDTA; 10 mM Hepes (pH 8) and pelleted by centrifugation at 15,000 rpm for 10 min. The unstacked membranes were solubilized with a final concentration of 0.6% α-DM; 10 mM Hepes (pH 8) at a chlorophyll concentration of 0.5 mg ml^−1^. Unsolubilized material was removed via 2 × centrifugation at 15,000 rpm for 30 min. The supernatant was loaded onto a Strep-Tactin gravity flow column (IBA), with all pre-made buffers being supplemented with 0.03% α-DM. The eluate was mixed with a sucrose buffer (2 M sucrose, 0.03% α-DM, 10 mM Hepes pH 7.5) to have a final sucrose concentration of 0.3 M. All procedures were performed at 4 °C in near darkness. Samples were aliquoted, snap-frozen in liquid N_2_ and stored at − 80 °C.

### Protein quantification, SDS-PAGE, immunoblotting, trypsin digest

PsbS protein concentration was determined via BCA assay (Pierce, Thermo Scientific). LHCII protein concentration was determined based on a Chl/protein (w/w) of 0.5. Chl concentration was determined using the method of^56^ but modified such that the absorption spectra are first fitted with the spectra of individual pigments^57^. SDS-PAGE was performed using a tris-tricine system with a 4% stacking and 12% running gel^58^. Immunoblotting was performed using antibodies from Agrisera. Images of gels/blots were taken with an ImageQuant LAS 4000 (GE). Relative protein levels were determined using Image Studio Lite (LI-COR). Enzymatic cleavage experiments were performed with an LHCII to Trypsin (from bovine pancreas; Merck) ratio of 50:1. The digest was carried out at 37 °C and stopped at 1 h with the addition of SDS-PAGE loading buffer and heating to 100 °C for 5 min.

### Proteoliposome preparation

Monogalactosyldiacylglycerol (MGDG), digalactosyldiacylglycerol (DGDG), sulphoquinovosyldiacylglycerol (SQDG) and l-α-phosphatidylglycerol (Soy PG) were purchased from Avanti Polar Lipids and suspended in 1:1 methanol:chloroform. A lipid mixture was prepared from a 40:30:15:15 molar ratio of MGDG, DGDG, SQDG and PG in a glass vial. Organic solvent was evaporated under N_2_ followed by 1 h under a low vacuum in a SpeedVac vacuum concentrator (Thermo Scientific). The lipid film was hydrated with either a 20 mM MOPS (pH 7.0) or 20 mM MES (pH 5.5) buffer containing 10 mM NaCl to give a final lipid concentration of 2.5 μg μL^−1^. The solution was vortexed for 1 h at RT. The hydrated lipids were then freeze-thawed 10× by alternating between liquid nitrogen and a 37 °C water bath before extrusion 10× through a 0.2-μm polycarbonate filter (Mini-Extruder, Avanti Polar Lipids). The liposomes were destabilized in 0.03% α-DM; 20 mM MOPS/MES (pH 7.5/5.5); 10 mM NaCl at a lipid concentration of 0.25 μg μL^−1^. The lipid-detergent mixture was equilibrated for 2 h at RT before being separated into three batches. Protein was added to the three batches according to the following lipid:protein molar ratios: (1) LHCII; 100:1 (2) LHCII; 133:1 (3) LHCII; 133:1 and PsbS; 400:1. Unless specified otherwise, the ratios refer to the LHCII monomer. Following addition of protein, the mixture was equilibrated for 30 min at RT. Absorbent beads (Bio-Beads SM2, Bio-Rad) were activated in methanol and rinsed thoroughly in hydration buffer before 20 mg mL^−1^ were added for 2 h and an additional 40 mg mL^−1^ for 30 min. During this time, samples were continuously mixed on an Eppendorf rotator at RT. Protein aggregates not incorporated into the liposome were sedimented by centrifugation at 15,000×*g* for 15 min. The proteoliposomes were subsequently stored at 4 °C and analyzed within 72 h.

### Steady-state spectroscopy

Absorptions spectra were recorded at RT on a 4000 UV–Vis-spectrophotometer (Varian). Fluorescence spectra were recorded at 77 K on a Fluorolog 3.22 spectrofluorimeter (Jobin Yvon-Spex). All fluorescence spectra were measured at an OD of < 0.05 cm^−1^ at the Q_y_ maximum. The circular-dichroism (CD) spectra were recorded on a Chirascan-Plus spectropolarimeter (Applied Photophysics). When necessary, the samples were diluted with a 20 mM MOPS (pH 7.5) or 20 mM MES (pH 5.5) buffer containing 10 mM NaCl. Plots were generated in OriginPro 2020 (OriginLab).

### Time-correlated single-photon counting (TCSPC)

Fluorescence decay kinetics were recorded on a FluoTime 200 fluorometer (PicoQuant). Excitation was provided by a 5 μW 468 nm laser diode with a repetition rate of 10 MHz. Fluorescence emission was detected at 680 nm. Samples were diluted to an OD of < 0.05 cm^−1^ at the Q_y_ maximum and stirred in a cuvette with a path length of 1 cm. All measurements were performed at 10 °C. Fluorescence decay curves were analyzed using FluoFit software (PicoQuant), following deconvolution of the instrument response function (IRF; 88 ps full width half maximum) measured from the ~ 6 ps decay of pinacyanol iodide‐dye dissolved in methanol^59^. The data were fitted to multi-exponential decay functions with amplitudes A_i_ and associated fluorescence decay times τ_i_. The average fluorescence lifetimes were calculated according to τ_avg_ = ΣA_i_ × τ_i_/ΣA_i._ Plots were generated in OriginPro 2020 (OriginLab).

## Supplementary Information

Supplementary Information

## Data Availability

The datasets and materials generated during and/or analysed during the current study are available from the corresponding author on reasonable request.
