# Supplementary material for: The PsbS protein and low pH are necessary and sufficient to induce quenching in the light-harvesting complex of plants LHCII
Source: Sci Rep. 2021 Apr 1;11:7415. doi: 10.1038/s41598-021-86975-9 (PMC8016914; doi:10.1038/s41598-021-86975-9)
Supplement: Supplementary file 1 — Supplementary Information [file 41598_2021_86975_MOESM1_ESM.pdf]

Supplementary information

The PsbS protein and low pH are necessary and sufficient to induce quenching in the light-harvesting complex of plants LHCII

Lauren Nicol[1] and Roberta Croce[1*]

[1] Biophysics of Photosynthesis, Department of Physics and Astronomy, Faculty of Sciences, Vrije Universiteit Amsterdam, Netherlands

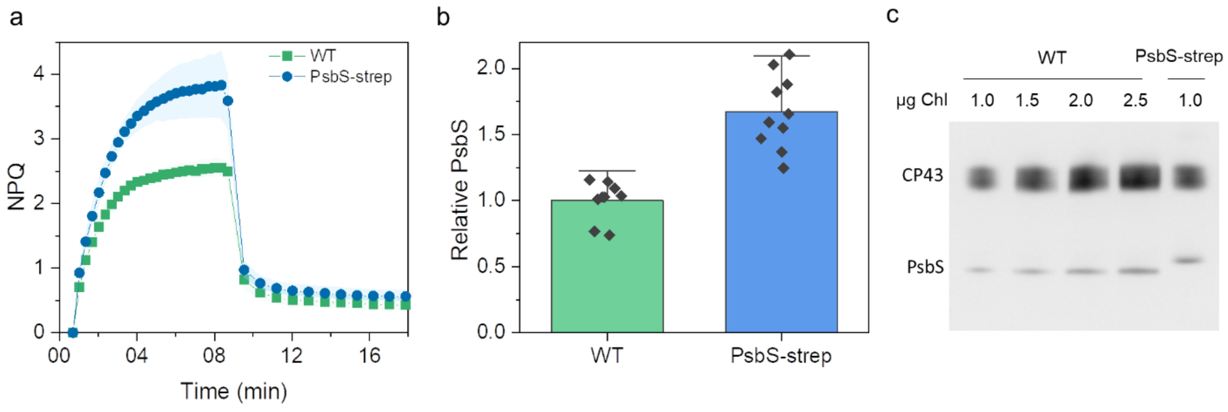

Figure S1. NPQ and PsbS levels of WT and PsbS-strep plants (a) NPQ kinetics of leaves when illuminated with 1,200 µmol photons m^{-2} s^{-1} for 8 minutes followed by a period of recovery in darkness. Data are mean ± s.d. (n = 5 biological replicas) (b) Relative PsbS levels based on densitometric analysis of immunoblots. PsbS signal intensities were normalized to the PSII core (CP43) and then to WT. Data are mean ± s.d. (n = 5 biological replicas). (c) Immunoblot of WT and PsbS-strep thylakoid membranes using antibodies against CP43 and PsbS.The full-length blot is presented in Supplementary Figure 4.

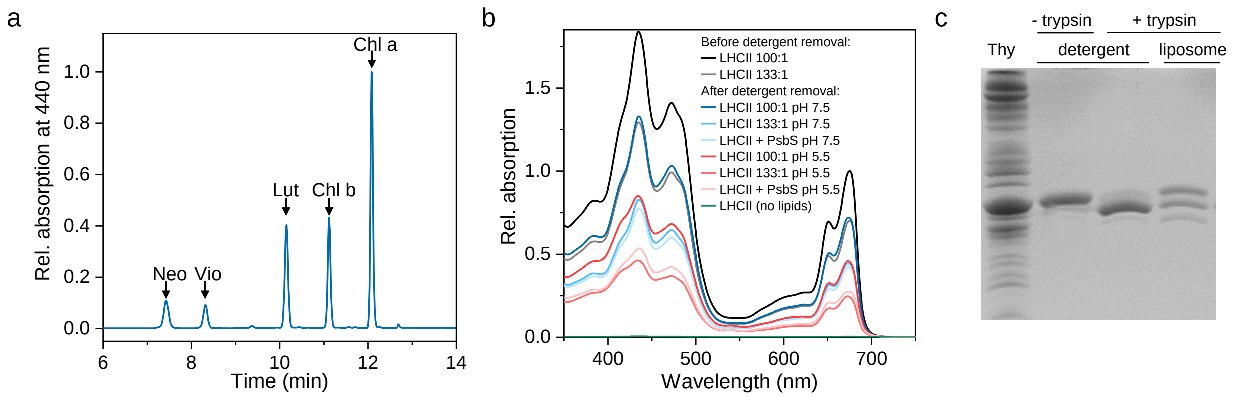

Figure S2. LHCII pigment composition, reconstitution efficiency and orientation in the liposome. (a) Chromatographic profiles of the pigments extracted from LHCII to indicate the presence of the three xanthophylls: Neo, neoxanthin; Vio, violaxanthin and Lut, lutein. (b) Absorption spectra of proteoliposome mixture before and after detergent removal and centrifugation to remove unreconstituted protein aggregates. (c) Coomassie-stained SDS-PAGE analysis of trypsin digest. From left to right: WT thylakoids, undigested LHCII in detergent, trypsin digested LHCII in detergent, trypsin digested LHCII in liposomes. Approximately 1 ug of LHCII is loaded in each lane. Trypsin cleaves the N-terminus of LHCII. The full-length gel is presented in Supplementary Figure 4.

Table S1. Fitting of fluorescence decay curves with exponential decay components

	**τ_1 (A_1)**	**τ_2 (A_2)**	**τ_3 (A_3)**	**τ_{avg}**
LHCII 0.03% α-DM	-	0.9 ns (12%)	3.6 ns (88%)	3.25 ns
LHCII 100:1 pH 7.5	-	0.9 ns (28%)	2.6 ns (72%)	2.14 ns
LHCII 133:1 pH 7.5	-	0.9 ns (26%)	2.8 ns (74%)	2.36 ns
LHCII + PsbS 100:1 pH 7.5	-	0.8 ns (20%)	2.7 ns (80%)	2.36 ns
LHCII 100:1 pH 5.5	0.2 ns (18%)	1.1 ns (29%)	2.5 ns (53%)	1.65 ns
LHCII 133:1 pH 5.5	0.2 ns (21%)	1.1 ns (31%)	2.6 ns (48%)	1.62 ns
LHCII + PsbS 100:1 pH 5.5	0.2 ns (17%)	0.8 ns (34%)	1.7 ns (49%)	1.11 ns

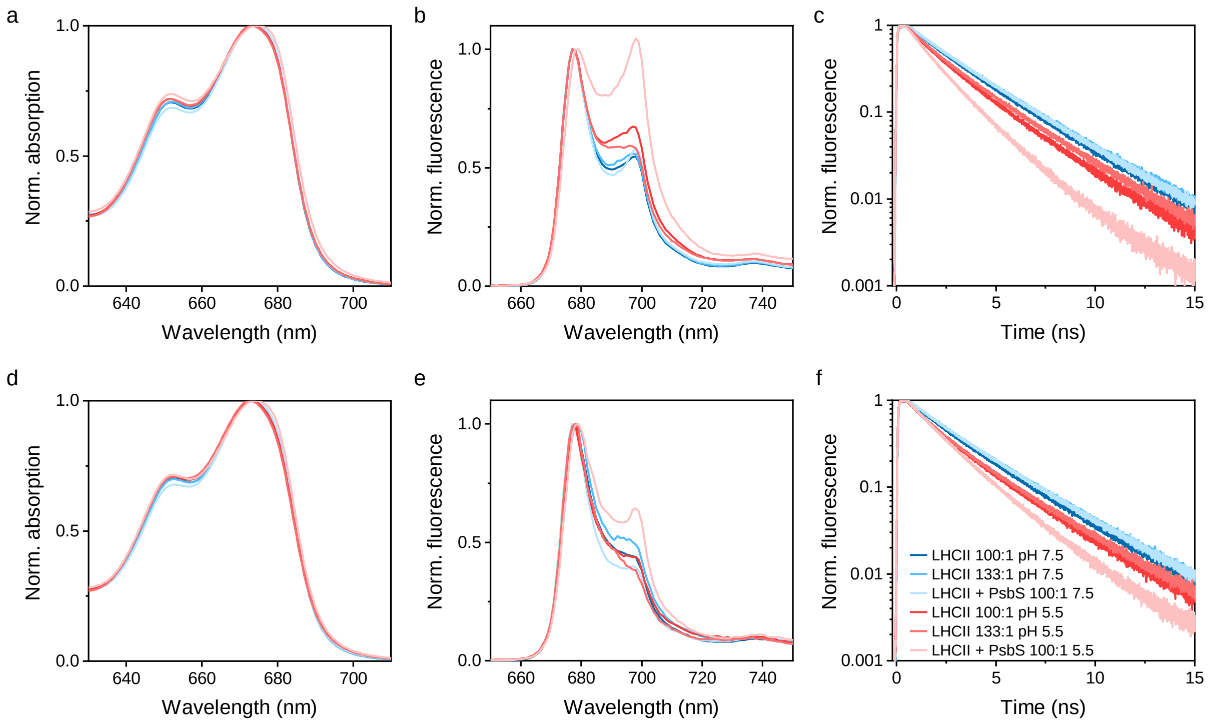

Figure S3. Two Independent replicas of proteoliposome spectral characteristics (a,d) absorption spectra normalized to the Q_y maximum (b,e) 77K fluorescence spectra upon excitation at 440nm, normalized to the maximum (c,f) fluorescence decay traces upon excitation at 468nm and detection at 680 nm. LHCII-only proteoliposome preparations have lipid:protein ratios of 100:1 and 133:1. LHCII + PsbS proteoliposome preparations have total lipid:protein ratios of 100:1, lipid:LHCII ratios of 133:1 and PsbS:LHCII ratios of 1:3.

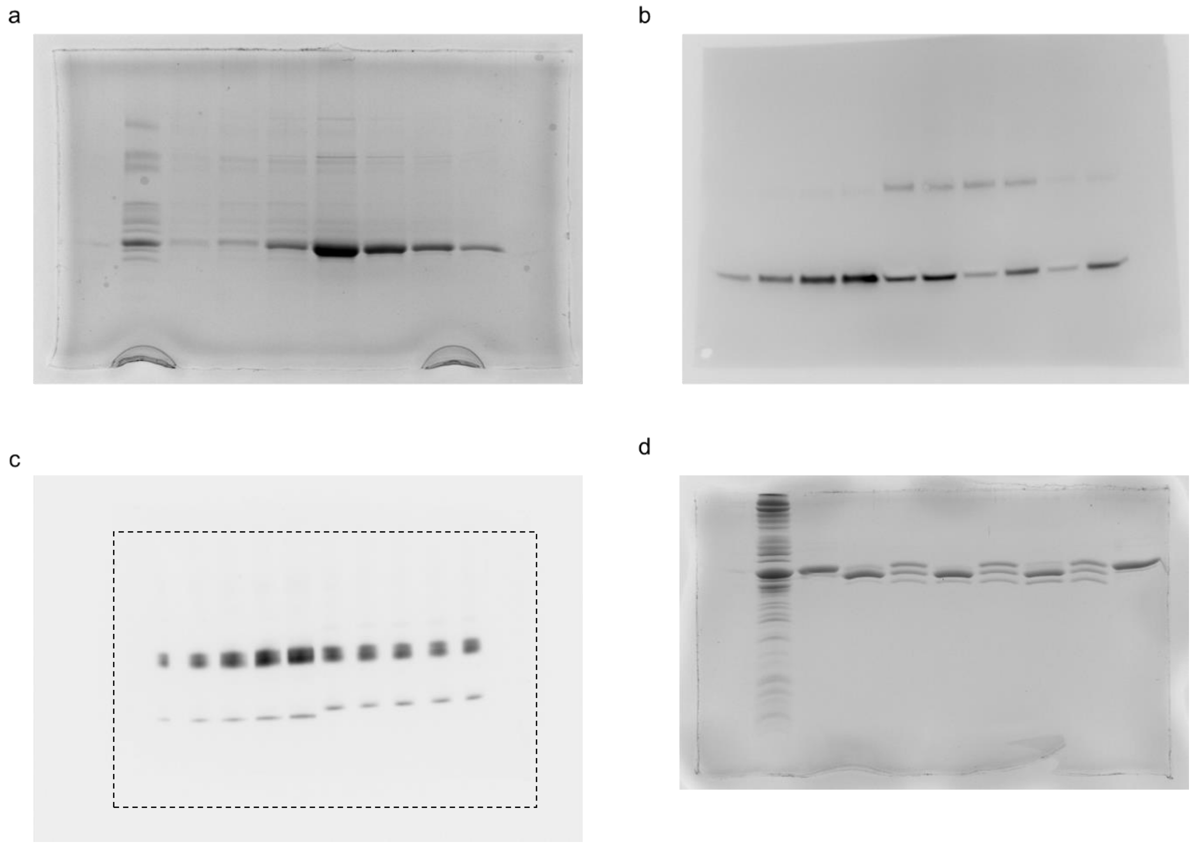

Figure S4. Full-length, unprocessed blots and gels. (a) SDS-PAGE gel from Figure 1. (b) Immunoblot of PsbS from Figure 3. (c) Immunoblot of PsbS and CP43 from Figure S1. (d) SDS-PAGE gel from Figure S2.
